# Synergistic remediation of Cd/Pb contamination in paddy soils using iron-based sulfur-rich material combined with foliar Zn fertilizer

**DOI:** 10.3389/fmicb.2025.1756253

**Published:** 2026-01-20

**Authors:** Ting Chen, Yuanyuan Sun, Fanxin Qin, Chunxiang Li, Lin Zhong, Jinjin Wang, Wanyu Huang, Haihe Wang, Qiufen Feng

**Affiliations:** 1 Key Laboratory of National Forestry and Grassland Administration on Biodiversity Conservation in Karst Mountainous Areas of Southwestern China, School of Life Sciences, Guizhou Normal University, Guiyang, China; 2 College of Natural Resources and Environment, Joint Institute for Environmental Research and Education, South China Agricultural University, Guangzhou, China; 3 Guizhou Institute of Environmental Scientific Research and Design, Guiyang, China; 4 Guizhou Ecological and Environment Monitoring Center, Guiyang, China; 5 Key Laboratory of Agro-Environment in Midstream of Yangtze Plain, Ministry of Agriculture, Hunan Cultivated Land and Agricultural Eco-Environment Institute, Changsha, China

**Keywords:** foliar spray, heavy metals, iron-based materials, microbial community, paddy soil, zinc

## Abstract

Addressing the limitations of single-technology approaches to mitigate cadmium (Cd) and lead (Pb) co-contamination in rice, this study elucidates the synergistic mechanism between iron-based soil immobilization and foliar zinc (Zn) barrier control. A pot experiment with four treatments was conducted: CK (control), FBA (foliar Zn fertilizer), IBS (iron-based sulfur-rich material), and ISF (IBS combined with Zn). The ISF treatment showed particularly remarkable efficacy. Compared to the CK, it increased rice biomass by 49.6%, reduced Cd and Pb concentrations in grains by 53.8 and 54.2%, respectively (to 0.2 mg/kg), and enhanced Zn accumulation by 311.8%. This treatment raised soil pH by 1.4 units, decreased bioavailable Cd and Pb by 31.2 and 18.5%, and promoted the transfomation of Cd into the Fe-Mn oxide-bound fraction (FMO, + 389.1%) and the residual fraction (RES, + 5.3%), while simultaneously increasing the FMO-bound proportion of Pb by 29.6%. Furthermore, ISF significantly enhanced soil enzyme activities (e.g., urease), increased total microbial OTUs with 187 unique OTUs, enriched *Nitrospirotaphyla*, *Desulfobacterota*, and *Geobacterales*, strengthened nitrogen/sulfur/iron cycling functions, and improved microbial network robustness. This research provides a theoretical foundation for heavy metal mitigation and nutritional fortification in rice production systems.

## 1 Introduction

The co-contamination of cadmium (Cd) and lead (Pb) presents a particularly severe threat in soils polluted by heavy metals. Characterized by high toxicity, persistence, and bioaccumulation potential, this contamination significantly endangers global agricultural product safety and ecosystem health (Xia et al., 2024; Sun et al., 2022; Wen et al., 2020). Cd and Pb are readily absorbed by crops and enter the food chain, causing irreversible damage to human renal, skeletal, and neurological systems (Tóth et al., 2016; Zhang et al., 2024). Rice serves as a staple food for half of the global population (Zhou et al., 2021). However, insufficient zinc (Zn) biofortification in this crop exacerbates hidden hunger. This deficiency further compromises immune function, while also impeding child growth and development (Wang X. J. et al., 2024). In China, intensified industrial and agricultural activities have led to excessive Cd/Pb levels in some croplands, severely constraining rice yield and nutritional quality enhancement. This has emerged as a critical barrier to sustainable agricultural development and has weakened the market competitiveness of agricultural products (Meng et al., 2019; Chen et al., 2025c).

The technique of *in situ* chemical immobilization is commonly utilized for the cleanup of soil contaminated with heavy metals, owing to its straightforward operation and economical nature (Li et al., 2014; Liu et al., 2021; Chen et al., 2025b). Traditional immobilizing agents, such as lime, apatite, clay minerals, and biochar, primarily reduce heavy metal bioavailability through adsorption, precipitation, or co-precipitation mechanisms (Li et al., 2025; Wei et al., 2025; Gao et al., 2019). However, single immobilizing agents often demonstrate insufficient efficiency for the simultaneous immobilization of Cd and Pb. Additionally, the long-term stability of immobilization effects is vulnerable to fluctuations in soil conditions, such as pH, organic matter, and redox potential (Lu et al., 2023; Lasisi et al., 2023; Yang et al., 2019). Furthermore, relying solely on soil immobilization inadequately controls heavy metal translocation to crop edible parts, resulting in limited efficacy for ensuring agricultural product safety.

To overcome the aforementioned limitations, a synergistic strategy that combines subterranean immobilization with phytophysiological inhibition in aerial parts shows promising potential. Regarding aerial parts, foliar Zn application effectively antagonizes Cd and Pb uptake and translocation. This occurs through competition for absorption sites and regulation of transporter expression (e.g., ZIP family proteins) (Zhong et al., 2024; Rodrigues et al., 2024). Additionally, it significantly improves crop growth status and enhances grain yield.

For subterranean remediation, iron-based materials have been demonstrated as efficient Cd/Pb immobilizing agents due to their strong adsorption capacity, surface complexation, and ability to induce iron oxide co-precipitate formation (Yin et al., 2017; Zhang et al., 2020; Lyu et al., 2018). Additionally, these materials are notable for their stability, nutrient retention capability, and the high reactivity of their iron constituents (Wan et al., 2022; Wang et al., 2025). Notably, resource-utilized Fe-rich sulfur-containing materials (e.g., modified industrial flue gas desulfurization byproducts) are enriched with reactive iron species such as FeOOH and Fe_3_O_4_ (Luo et al., 2025). These materials exhibit advantages of high specific surface area and low cost (Chen et al., 2025a).

Their application in soil remediation achieves environmental co-benefits through the principle of “using waste to treat contamination,” while providing abundant reactive iron sources. Although the individual effects of foliar Zn application have been studied, the impacts of soil iron-based material amendment have also been examined separately. However, the comprehensive inhibitory effects of their synergy on Cd and Pb migration and accumulation in soil-rice systems remain unclear. The underlying mechanisms behind this synergy are still not fully understood. Crucially, the efficacy and mechanistic role of resource-utilized iron-rich sulfur-containing materials within this synergistic system have yet to be elucidated.

Soil microbial communities serve as key biological indicators for evaluating soil health (Wang F. et al., 2024; Tang et al., 2022). Heavy metal contamination not only directly alters microbial composition and diminishes functional diversity but also hampers critical nutrient cycling and pollutant detoxification processes. Compared to fungi, bacteria show greater sensitivity to heavy metal stress (Rieder and Frey, 2013; Stefanowicz et al., 2010), rendering them as early-warning indicators for responses to remediation efforts. Soil enzyme activities, such as those of phosphatase and peroxidase, quantify the efficiency of microbe-mediated nutrient cycling and detoxification capacity (Siczek and Lipiec, 2016; Wang et al., 2021; Bellotti et al., 2022). Microbial diversity underpins enzyme synthesis, while enzyme activity directly mirrors the intensity of functional output. Together, they unveil soil response mechanisms under contamination stress and the efficacy of remediation measures (Yang et al., 2022).

Based on this, our study systematically evaluates the impacts of foliar Zn application and soil-applied iron-based materials on key processes within the soil-rice system. This evaluation is conducted through integrated pot experiments. These experiments combine chemical analysis with high-throughput sequencing techniques. These processes include: (1) rice physiological responses and the accumulation of Cd, Pb, and Zn in various tissues; (2) the evolution of soil pH and the dynamic changes in Cd and Pb fraction; and (3) the responses of microbial community structure and function, including enzyme activities. This study aims to elucidate the synergistic mechanism of “aboveground Zn blocking and underground Fe-based immobilization,” thereby providing a scientific basis for developing efficient farmland remediation strategies.

## 2 Materials and methods

### 2.1 Experimental materials

The paddy soil was sourced from the upper 0–20 cm layer of a rice paddy in Guiyang City, Guizhou Province (27°12′0″N, 107°13′10″E), exhibiting a pH of 7.03 (Supplementary Table 1). The total Cd and Pb contents in the soil were 1.31 and 292.39 mg/kg, respectively, significantly surpassing the agricultural risk screening values stipulated by the National Soil Environmental Quality Standard (GB 15618-2018) (Cd: 0.3 mg/kg, Pb: 120 mg/kg). Based on multi-year field screening results in Kaiyang, the rice cultivar Chuankangyou 6107, known for its high Cd/Pb accumulation characteristics, was selected for pot experiments (Chen et al., 2025c). Amendment materials included: (1) Foliar Zn fertilizer (Zn ≥ 160 g/L) purchased from Qingdao Yasefu Trading Co., Ltd.; (2) The iron-based sulfur-rich material, supplied by Hunan Iron & Steel Group (Changsha) with a pH of 10.7, has been classified as an environmentally friendly material through safety assessment (Feng et al., 2022) and requires sieving through a 0.149-mm mesh prior to use.

### 2.2 Pot experiment design

This study was conducted from May to October 2022 in the greenhouse of Guizhou Normal University (106°37′E, 26°23′N, altitude 1155.5 m) using a pot experiment. Each pot contained 3 kg of soil sieved through a 2-mm mesh. Soil amendments were uniformly mixed into the soil at a rate of 1% (w/w). Subsequently, the soil surface was flooded to a depth of 3 cm with water and maintained under outdoor flooded conditions for 1 month. Basal fertilizers comprising urea (0.2 g/kg) and ammonium dihydrogen phosphate (0.1 g/kg) were mixed into the soil 1 week before transplanting, with dosages calculated per kg of soil. The seeds underwent surface sterilization using 5% hydrogen peroxide for a duration of 20 min, followed by a thorough rinse with deionized water, and were subsequently arranged on moist filter paper within Petri dishes. Germination was induced by incubation at 28°C in a plant growth chamber (LRH-1000A-GSIE, China) under dark conditions for 48 h. The resulting seedlings were transferred to vermiculite nursery trays and regularly supplied with nutrient solution until reaching the four-leaf stage. Uniform seedlings were selected and transplanted into pots at a density of two hills per pot and three seedlings per hill. Four treatments were established: CK (control), FBA (foliar Zn fertilizer), IBS (iron-based sulfur-enriched material), and ISF (IBS combined with Zn).

During the tillering phase, 1.2 mL of Zn-based foliar fertilizer stock solution was diluted directly to 800 mL. For the heading phase, the volume was doubled to 2.4 mL and diluted to 1,600 mL. Prior to spraying, the soil surface in each pot was tightly covered with plastic film to prevent solution contact. At the tillering stage, 100 mL of the diluted solution was sprayed per pot; at the heading stage, 200 mL was sprayed per pot. Spraying was conducted until fine droplets uniformly covered the leaf surfaces. After natural drying, the plastic film was removed. The CK was synchronously sprayed with an equivalent volume of deionized water. Throughout the experimental period, pot positions were rotated regularly, and the soil was loosened periodically to maintain soil moisture at approximately 70% of the field capacity.

### 2.3 Samples selection and collection

After cultivating rice for 120 days, the entire plants were harvested, with fresh rhizospheric soil adhering to the roots. A sample of this soil was stored at −80°C in an ultra-low freezer (DW-HL340, China) for the purpose of microbial analysis, while the remainder was air-dried, ground, and sieved for the analysis of physicochemical properties and heavy metal fractions. Productive panicle numbers and grain yields were recorded prior to washing the plants with tap water and rinsing them three times with deionized water. Plant height and root length were measured, and the plants were separated into roots, stems, and leaves. Initially, the tissue samples were deactivated in a drying oven (DZF-6050, China) at 105°C for a duration of 30 min, followed by drying them at 75°C until a constant weight was achieved. Finally, the samples were ground using a grinder (BJ-800A, China). The grains were air-dried and dehulled using a brown rice sheller (JLGJB-45, China) to separate the brown rice from the husks. Both the brown rice and husks were dried at 60°C and ground. Ground samples of brown rice, husks, roots, stems, and leaves were stored separately in zip-lock bags for future chemical analysis.

### 2.4 Experimental methods

A 10.00 g soil sample was added to a 50 mL beaker and combined with 25 mL of distilled water that was free of CO_2_ (with a soil-to-water ratio of 1:2.5, w/w). The mixture was stirred vigorously for 1 min and then allowed to settle for 30 min. The pH of the resulting supernatant was determined using a pH meter (S210, China). Cd, Pb, and Zn concentrations in soil and rice tissues were quantified by inductively coupled plasma mass spectrometry (ICP-MS, ICAP RQ, United States), following acid digestion. For soil digestion, 0.2000 g of a 100-mesh sieved sample was accurately weighed into a polytetrafluoroethylene (PTFE) crucible, digested on a hotplate with sequential additions of 7 mL HNO_3_, 5 mL HF, and 5 mL HClO_4_ until complete dissolution, cooled, diluted to 50 mL, centrifuged, and the supernatant collected for analysis. Rice plant digestion involved weighing 0.2000 g of powdered sample into a digestion tube, adding 10 mL of HNO_3_-HClO_4_ mixed solution (9:1, v/v), capping and soaking overnight, then heating on a hotplate until the digestate turned colorless or pale yellow, cooling, and diluting to 25 mL with 1% HNO_3_. Bioavailable Cd and Pb in soil were extracted using the DTPA-CaCl_2_-TEA buffer according to Chinese National Standard HJ 804-2016, with extracts analyzed by ICP-MS. Chemical fractions of Cd/Pb in rhizosphere soil was performed using the Tessier five-step sequential extraction procedure (Wang M. et al., 2024; Supplementary Figure 1). Soil enzyme activities were measured according to Kong and Wu (2012): alkaline phosphatase (S-AKP) activity via disodium phenyl phosphate colorimetry; urease (S-UE) activity via indophenol blue colorimetry; dehydrogenase (S-DHA) activity via optimized TTC reduction; catalase (S-CAT) activity via potassium permanganate titration.

### 2.5 Bacterial community profiling

DNA was isolated from soil microorganisms employing the E.Z.N.A.^®^ Soil DNA Kit (Omega Bio-tek, United States). Subsequent evaluation of DNA quality involved 1% agarose gel electrophoresis, with concentration and purity quantification performed on a NanoDrop 2000 spectrophotometer (Thermo Scientific, United States). Amplification of the 16S rRNA gene V3-V4 region utilized barcoded primers 515F (5′-GTGCCAGCMGCCGCGG-3′) and 907R (5′-CCGTCAATTCMTTTRAGTTT-3′), using total DNA as the template. The resultant PCR amplicons were recovered by 2% agarose gel electrophoresis, cleansed via a DNA Gel Extraction Kit (PCR Clean-Up Kit, China), and precisely measured using a Qubit 4.0 Fluorometer (Thermo Fisher Scientific, United States).

Library preparation was performed with purified amplicons using the NEXTFLEX^®^ Rapid DNA-Seq Kit (Bioo Scientific, United States). Pair-end sequencing was conducted on an Illumina NextSeq 2000 system (Illumina, United States) through Majorbio BioPharm Technology Co., Ltd. (China). Raw paired-end reads underwent quality control via fastp (v0.19.6) (Chen et al., 2018) and were assembled with FLASH (v1.2.11) (Magoč and Salzberg, 2011). After quality filtering, sequences were clustered into operational taxonomic units (OTUs) at 97% sequence similarity followed by chimera removal. To normalize sequencing depth, all samples were rarefied to 20,000 sequences per sample (post-rarefaction Good’s coverage averaging 99.09%). Taxonomic classification of OTUs was executed using the RDP classifier (v2.11) (Wang, 2007) against the SILVA 16S rRNA database (release 138) with a 70% confidence cutoff, and community composition was evaluated across multiple taxonomic ranks.

Alpha diversity indices under rarefied sampling were calculated using mothur (v1.48.0) (Schloss et al., 2009). Community structural similarity was evaluated through Principal Coordinate Analysis (PCoA) employing Bray-Curtis distances, and intergroup divergences were assessed for statistical significance using PERMANOVA with 999 permutations. Sample-species relationships were visualized using Circos. Significantly differential taxa from phylum to order level were identified by LEfSe (LDA > 2, α = 0.05) (Segata et al., 2011). Spearman correlations between environmental factors and phylum-level abundances (|R| > 0.5, *P* < 0.05) were computed using the Hmisc (v5.2.3) package; correlation networks were constructed via ggraph (v2.2.2) and node importance evaluated by degree centrality. Microbial co-occurrence networks were constructed in Gephi (v0.10.1) employing the Fruchterman-Reingold layout algorithm with correlation thresholds of |R| > 0.8 and *P* < 0.001, followed by quantification of topological properties and network robustness analysis with ggClusterNet (v2.0). Bacterial functional profiles were ultimately predicted using FAPROTAX.

### 2.6 Computational and statistical approaches

Data were organized in Microsoft Excel 2021 (Microsoft Corp., United States). Results are expressed as mean ± SD. Statistical comparisons were performed using one-way ANOVA followed by LSD *post hoc* tests (*P* < 0.05) in IBM SPSS Statistics 27 (IBM Corp., United States), with distinct lowercase letters designating significant differences in multiple comparisons. Data visualization was conducted with Origin 2022 (OriginLab Corp., United States) and GraphPad Prism 10.4.0 (GraphPad Software, United States).

## 3 Results

### 3.1 Rice growth and its contents and transfer coefficients of Cd, Pb, and Zn

**FIGURE 1:**
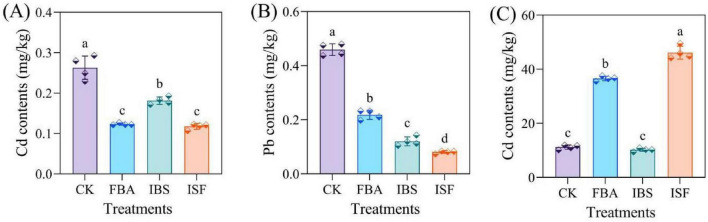
Concentrations of **(A)** Cd, **(B)** Pb, and **(C)** Zn in rice grain following treatments with FBA, IBS, and ISF. Distinct letter superscripts denote statistical significance (*P* < 0.05). Data are expressed as mean ± SD (*n* = 4).

Compared with the CK, the rice yield of the FBA, IBS, and ISF treatment groups increased by 12.1–49.6%, the number of productive tillers by 15.1–30.1%, and root length by 3.9–23.7%, with the ISF group showing the most significant increase. Conversely, plant height decreased by 6.36–6.75% (*P* < 0.05) (Supplementary Figure 2). The concentrations of Cd and Pb in rice grains decreased by 30.8–53.8% and 16.7–54.2% across all treatments, respectively (Figures 1A,B). Both the IBS and ISF groups exhibited Cd and Pb levels below the limits specified in GB 2762-2022. Notably, the ISF treatment increased Zn accumulation in rice grains by 311.8% (Figure 1C).

Analysis of element translocation pathways (Supplementary Figure 3) indicated reduced translocation factors (TFs) for Cd and Pb across all treatment groups. These reductions occurred both from roots to stems/leaves and from stems/leaves to grains. All experimental groups (FBA, IBS, ISF) showed lower TFs compared to the CK group. Notably, the Cd TF from stems/leaves to husks increased in the FBA group, whereas it decreased in the IBS and ISF groups. In contrast, the Pb TF from stems/leaves to husks showed an increasing trend in all three treatments. The Zn TF from roots to stems and leaves, as well as from stems and leaves to husks and grains, was significantly higher in the FBA and ISF treatments.

### 3.2 Soil Cd/Pb content and fractions distribution

**FIGURE 2:**
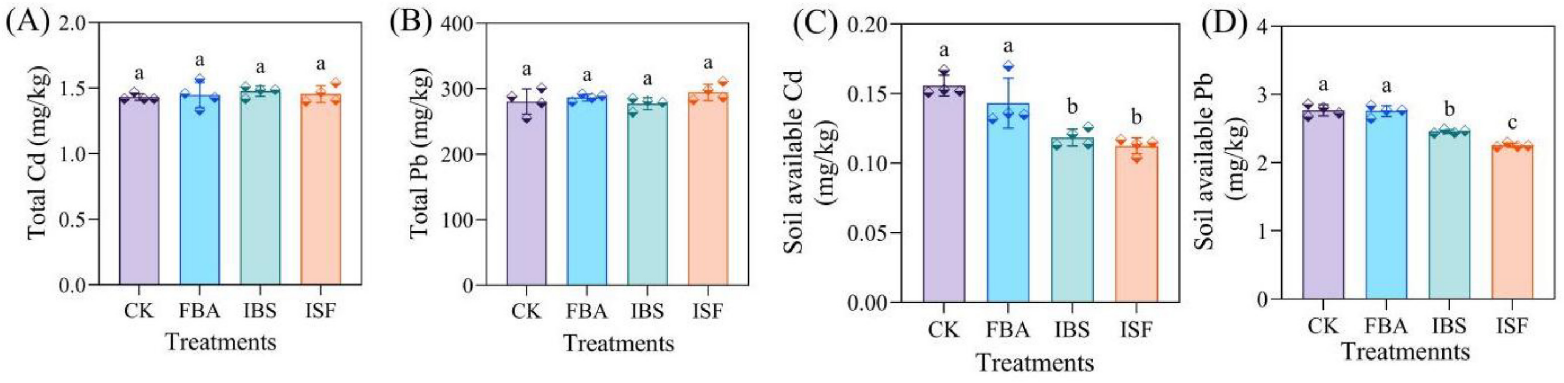
Changes in total **(A,B)** and available **(C,D)** fractions of Cd and Pb in soil amended with FBA, IBS, and ISF. Distinct letter superscripts denote statistical significance (*P* < 0.05). Data are expressed as mean ± SD (*n* = 4).

**FIGURE 3:**
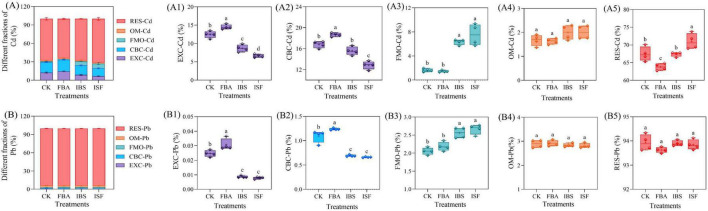
Chemical fractions of **(A)** Cd and **(B)** Pb in soils amended with FBA, IBS, and ISF: **(A1,B1)** EXC: Exchangeable, **(A2,B2)** CBC: Carbonate-bound, **(A3,B3)** FMO: Fe/Mn oxide-bound, **(A4,B4)** OM: Organic matter-bound, **(A5,B5)** RES: Residual. Distinct letter superscripts denote statistical significance (*P* < 0.05). Data are expressed as mean ± SD (*n* = 4).

Compared with the CK, no significant differences (*P* > 0.05) were detected in the total Cd/Pb content of soil among the FBA, IBS, and ISF treatment groups (Figures 2A,B). The bioavailable Cd fraction decreased by 12.5–31.2%, and the bioavailable Pb fraction by 0.4–18.5% (Figures 2C,D). Notably, the ISF and IBS groups exhibited significantly greater reductions in bioavailable fractions than the FBA group (*P* < 0.05). Tessier sequential extraction (Figure 3) revealed that the IBS and ISF treatments resulted in lower levels of exchangeable (EXC) and carbonate-bound (CBC) Cd/Pb fractions, while the Fe/Mn oxide-bound (FMO) Cd fraction increased by 389.1% and Pb by 29.6%, with no significant change in the organic matter-bound (OM) fraction. The residual (RES) Cd fraction increased by 5.3%, whereas the residual Pb fraction showed no significant change.

### 3.3 Soil pH and correlation analysis

**FIGURE 4:**
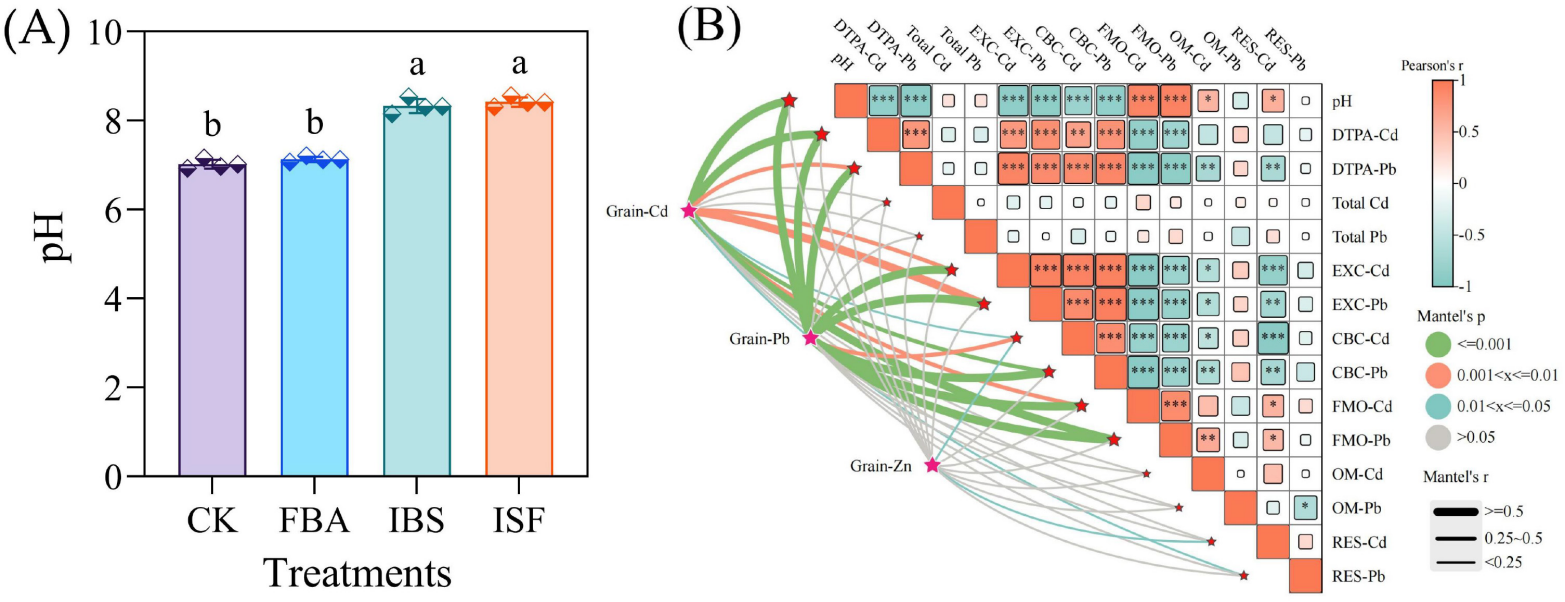
**(A)** pH values in amended soils. **(B)** Interactive Mantel test correlation heatmap: EXC, Exchangeable; CBC, Carbonate-bound; FMO, Fe/Mn oxide-bound; OM, Organic matter-bound; RES, Residual. Revealing interrelationships among grain Cd, Pb, and Zn content, environmental factors: Edge width corresponds to the strength of correlation represented by the absolute value of Mantel’s r (|R|). Color gradient: red signifies positive, blue signifies negative. Pearson significance levels: *P ≤ 0.05, **P ≤ 0.01, ***P ≤ 0.001 (*n* = 16). Distinct letter superscripts denote statistical significance (*P* < 0.05). Data are expressed as mean ± SD (*n* = 4).

The soil pH in iron-based material treatments (IBS and ISF) increased by 1.3–1.4 units compared to the CK (Figure 4A). Pearson correlation analysis (Figure 4B) demonstrated highly significant negative associations (*P* ≤ 0.001) between soil pH and DTPA-extractable/EXC-Cd/Pb concentrations, along with significant negative correlations (*P* ≤ 0.05) for CBC-Cd/Pb. Conversely, soil pH exhibited significant positive correlations (*P* ≤ 0.05) with FMO-Cd/Pb, OM-Cd, and RES-Cd. Moreover, highly significant positive correlations (*P* ≤ 0.001) were observed between EXC-Cd/Pb and CBC-Cd/Pb, whereas both EXC and CBC fractions showed significant negative correlations (*P* ≤ 0.05) with FMO-Cd/Pb, OM-Cd, and RES-Cd. Mantel tests further confirmed that both soil pH and Cd/Pb speciation significantly influenced Cd and Pb accumulation in grains (*P* ≤ 0.05; R > 0.25), but had no significant effect on grain Zn content.

### 3.4 Soil enzyme activity

**FIGURE 5:**
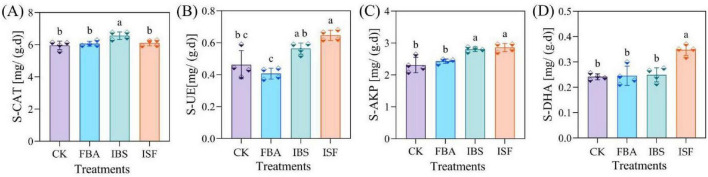
Effects of FBA, IBS, and ISF on the activities of **(A)** catalase (S-CAT), **(B)** urease (S-UE), **(C)** alkaline phosphatase (S-AKP), and **(D)** dehydrogenase (S-DHA). Distinct letter superscripts denote statistical significance (*P* < 0.05). Data are expressed as mean ± SD (*n* = 4).

Compared with the CK, the ISF treatment significantly enhanced the activities of soil S-UE, S-AKP, and S-DHA (*P* < 0.05), with increases of 39.1, 23.%, and 45.8%, respectively. However, it had no significant effect on S-CAT activity (Figure 5). In contrast, the IBS treatment significantly increased S-CAT activity by 10.3%. The FBA treatment significantly reduced S-UE activity by 10.9%, while showing no significant effects on S-AKP, S-DHA, or S-CAT activities. Overall, ISF exhibits a pronounced promoting effect on the activities of S-UE, S-AKP, and S-DHA.

### 3.5 Impact of ISF treatment on the rhizosphere microbial community

The read-coverage-based rarefaction curve (Supplementary Figure 4) indicated that the soil bacterial sequencing depth approached saturation at approximately 4,000 sequences. Good’s coverage indices exceeded 0.96 across all samples (Supplementary Table 2). These results demonstrate sufficient sequencing depth to capture the majority of microbial diversity.

**FIGURE 6:**
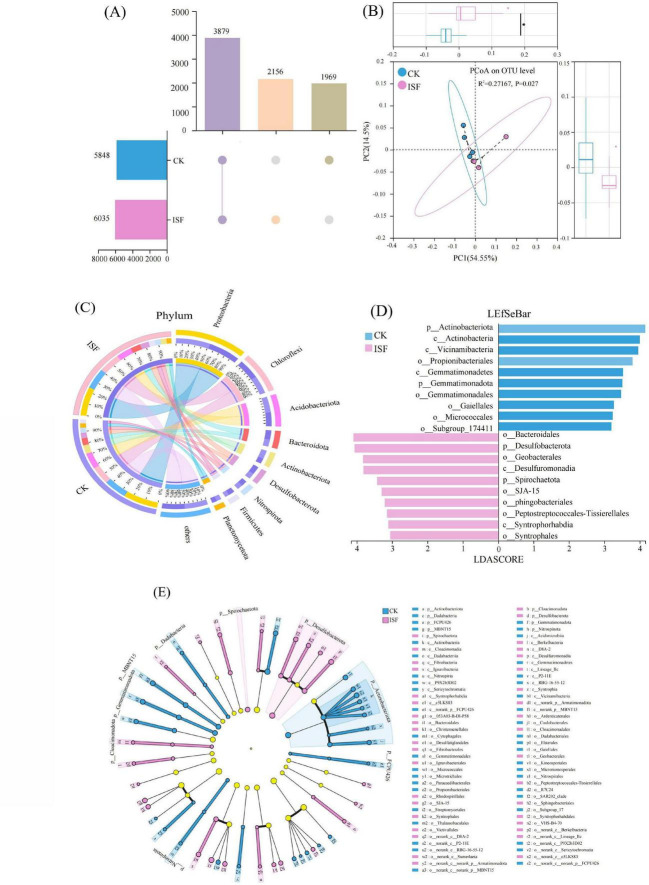
Effects of ISF on rhizosphere soil bacterial community structure and composition: **(A)** Intersection analysis of bacterial taxa (Upset plot). **(B)** Beta diversity (Principal component analysis, PCoA). **(C)** Taxon abundance distribution (Circos diagram). **(D,E)** Biomarker identification (LEfSe). Asterisk denotes significant difference relative to the control group: *P < 0.05.

Alpha diversity analysis revealed no statistically significant differences (*P* > 0.05) in Chao1, Shannon, or Simpson indices between the ISF treatment and the CK. At the OTU level, the ISF treatment group displayed a higher total OTU count compared to the control and harbored 187 unique additional OTUs (Figure 6A).

Based on beta diversity analysis, further examination was conducted using PCoA and PERMANOVA testing (Figure 6B). The PERMANOVA results revealed an R2 value of 0.27 (*P* = 0.02). In PCoA, the contribution rates of the principal coordinate (PC) axes 1 and 2 were 54.55 and 14.50%, respectively, which were associated with the compositional distribution of CK and ISF communities.

Analysis of community structure (Figure 6C) indicated significant changes between ISF treatment and CK group. The ISF treatment increased the relative abundances of *Desulfobacterota* and *Nitrospirota* phyla. Conversely, it decreased the proportions of *Acidobacteriota* and *Actinobacteriota* phyla. Notably, the CK group showed a significantly higher abundance of *Actinobacteriota*. The LEfSe analysis (Figure 6D) identified *Bacteroidales* (LDA = 4.09), *Desulfobacterota* (LDA = 4.06), and *Geobacterales* (LDA = 3.82) as indicator taxa for the ISF group, whereas the CK group was characterized by *Actinobacteriota* (LDA = 4.15).

**FIGURE 7:**
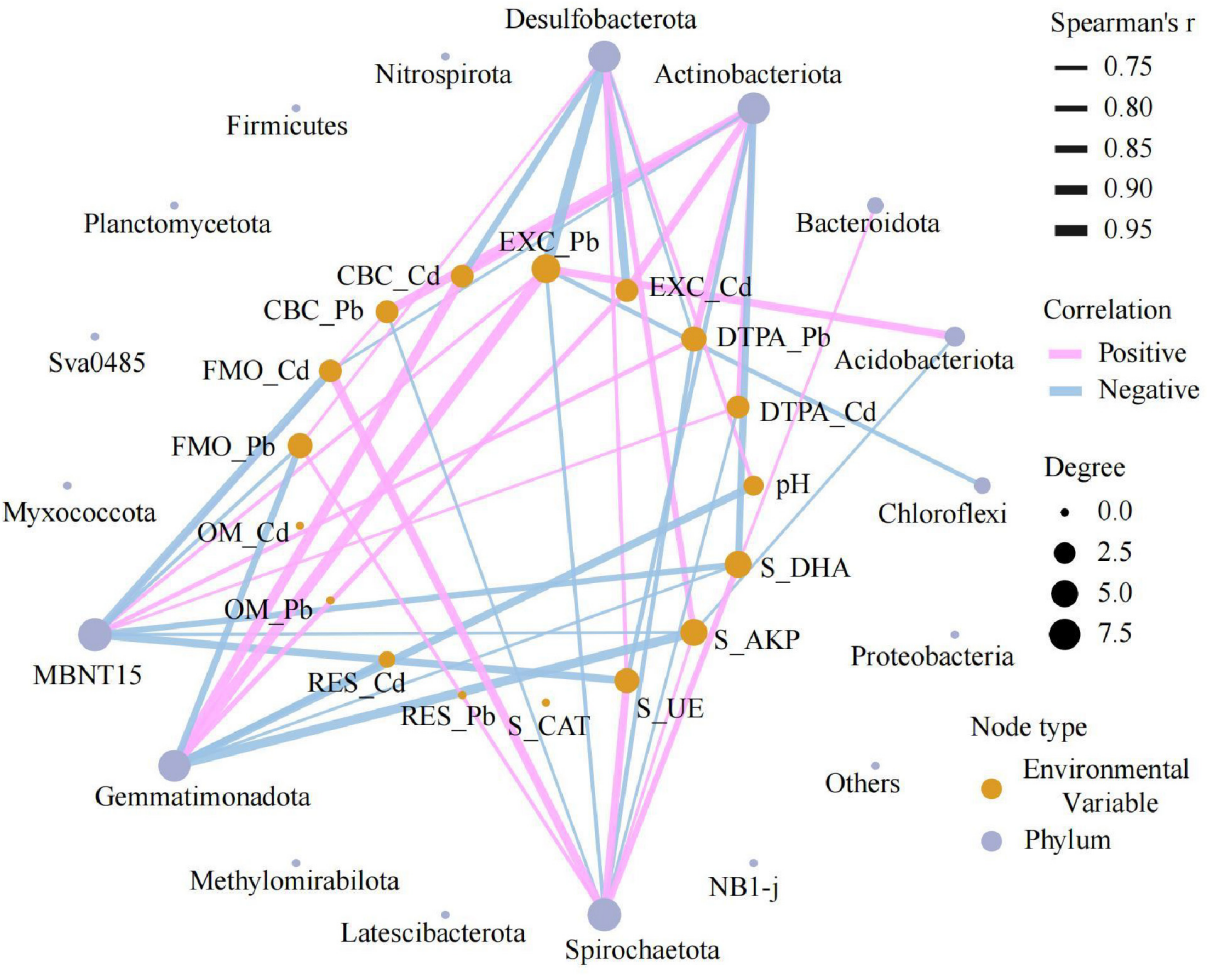
Co-occurrence network between environmental factors and bacterial phylum-level communities.

Phylogenetic tracing (Figure 6E) indicated that *Desulfobacterota* (δ-*proteobacteria*) enriched in ISF were predominantly distributed within *Syntrophorhabdales* and *Geobacterales* orders. In contrast, *Actinobacteriota* (*Actinobacteria*) enriched in CK exhibited distinct taxonomic distributions. Co-occurrence network analysis (Figure 7) revealed that *Desulfobacterota* had positive correlations with FMO-Pb, S-UE, S-AKP, and pH (*P* < 0.05), but negative correlations with CBC-Cd/Pb and DTPA-Pb (*P* < 0.05).

### 3.6 FAPROTAX and microbial co-occurrence networks

**FIGURE 8:**
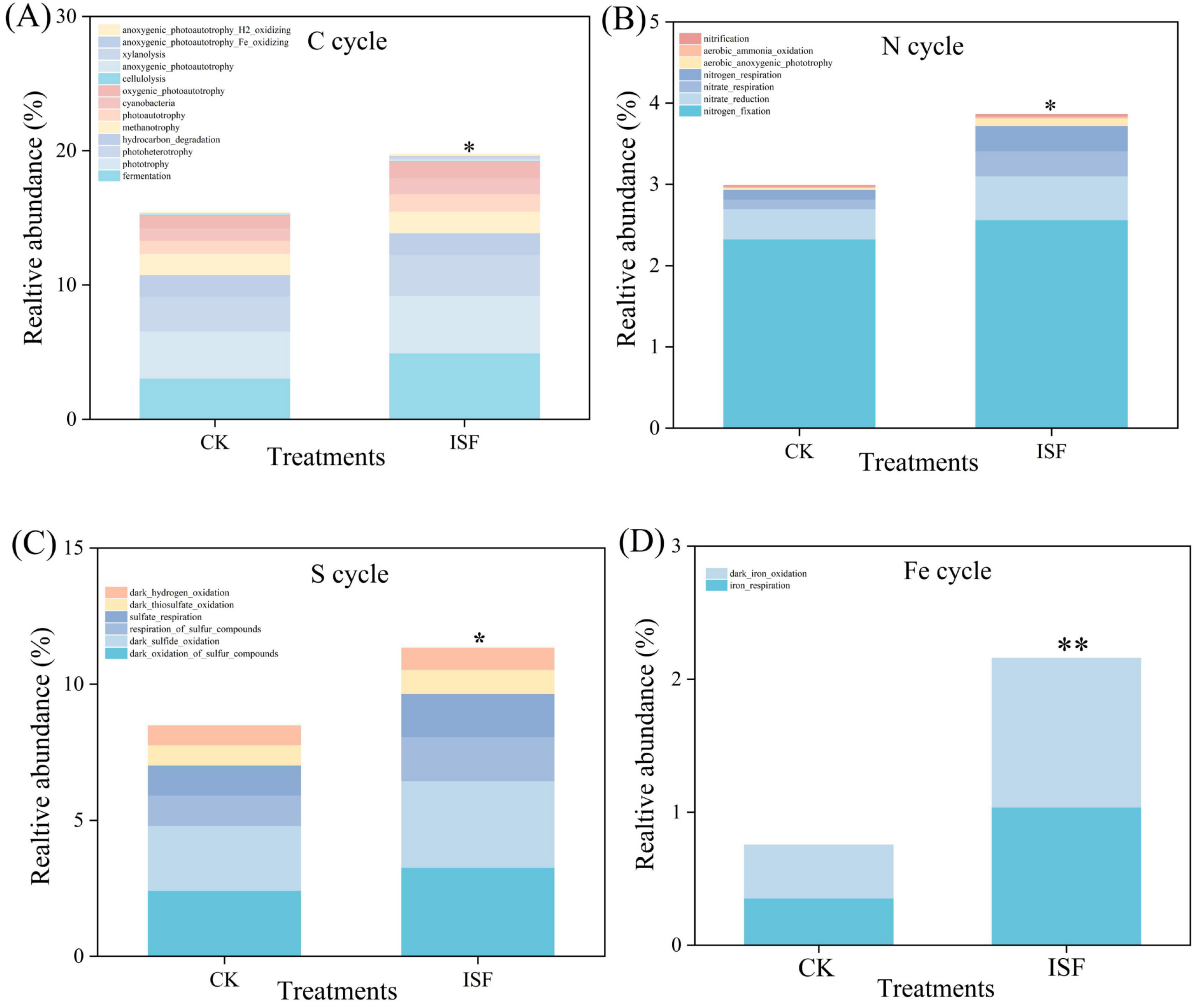
Variation of the main ecological functions profiles of bacterial communities by FAPROTAX. Relative abundance of **(A)** carbon (C) cycle, **(B)** nitrogen (N) cycle, **(C)** sulfur (S) cycle and **(D)** iron (Fe) cycle. Asterisk denotes significant difference relative to the control group: *P < 0.05, **P < 0.01.

The ecological functional analysis of soil bacteria, utilizing FAPROTAX (Figure 8), revealed that ISF significantly improved carbon (C) cycling functions (*P* < 0.05) compared to the CK. This enhancement particularly included fermentation, oxygenic photoautotrophy, and hydrocarbon degradation. Additionally, ISF substantially boosted nitrogen (N) fixation, nitrate reduction, and nitrate respiration within the nitrogen cycle (*P* < 0.05), and intensified dark oxidation of sulfur compounds, sulfur respiration, and sulfate respiration in the sulfur (S) cycle (*P* < 0.05). Notably, the stimulation effect on iron respiration within the iron (Fe) cycle was particularly significant (*P* < 0.01).

**FIGURE 9:**
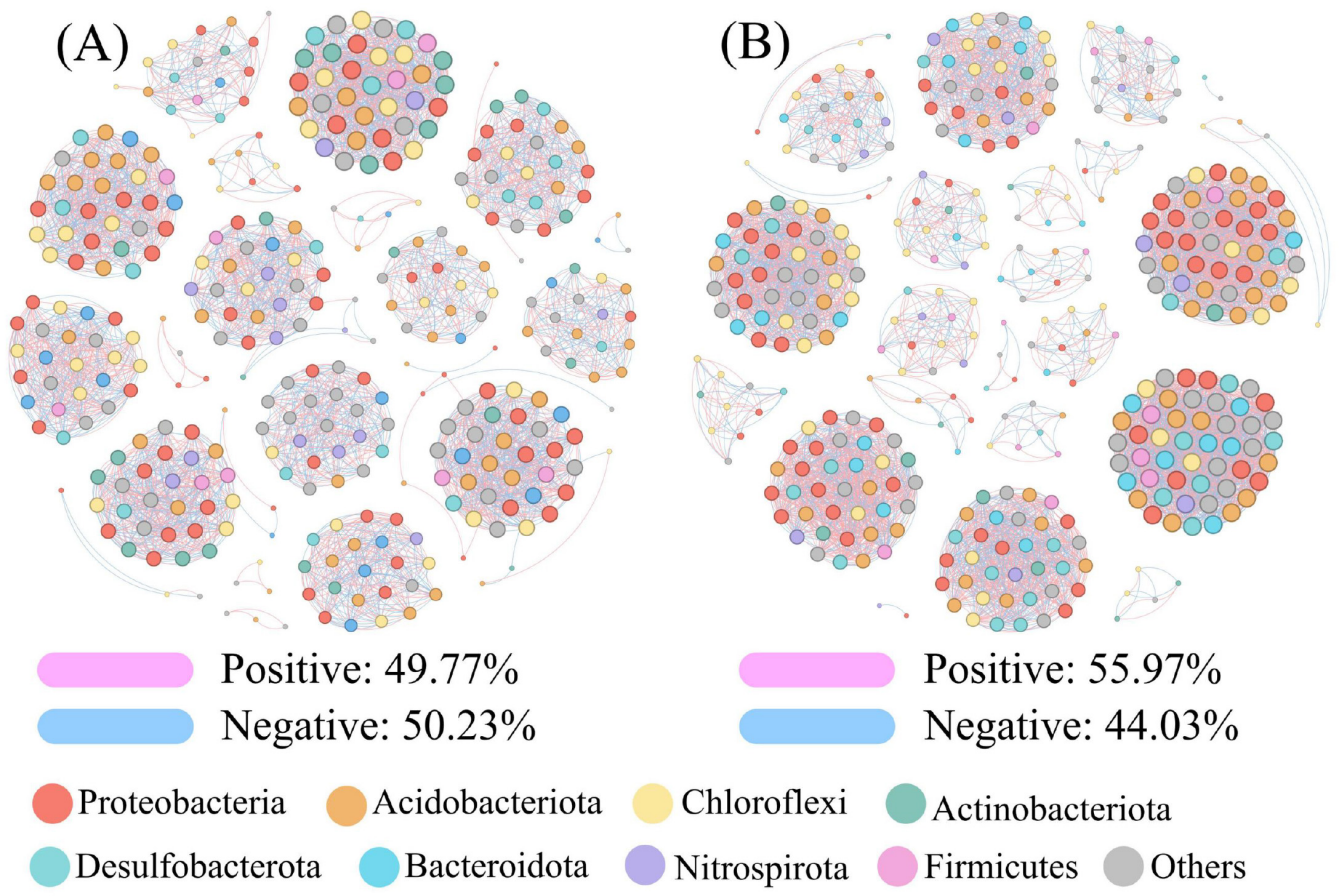
Microbial association networks: **(A)** CK, **(B)** ISF.

The analysis of the bacterial association networks revealed that, compared to the CK group (344 nodes, 3751 edges, with 49.77% positive edges), the ISF treatment group showed an increase in the number of nodes, the number of edges, the average degree, and the network density (Supplementary Table 3). Both networks exhibited high modularity (modularity index > 0.85; CK group: 0.899) and a similar composition of dominant bacterial phyla (Figures 9A,B). In the robustness analysis (Supplementary Figure 5), both ISF and CK groups showed identical absolute linear regression slope values of −0.001. However, the coefficient of determination (R^2^) was higher in the ISF group (0.821) than in the CK group (0.021).

## 4 Discussion

### 4.1 ISF drives the transformation of Cd/Pb into stable fractions through pH modulation

Previous studies (Lan et al., 2020; Umar et al., 2025; Wang F. et al., 2024) indicate that the success criterion for remediating heavy metal-contaminated farmland soil lies in significantly reducing heavy metal content in crops. This remediation must simultaneously maintain or improve crop yield and quality. This study revealed that ISF treatment significantly reduced the concentrations of Cd and Pb in rice grains by 65.3 and 54.2%, respectively (Figures 1A,B), bringing them below the national safety standards. Concurrently, the rice yield increased by 49.6% (Supplementary Figure 2). These effects were achieved through the optimization of soil physicochemical properties, the reshaping of the microbial community, and synergistic regulation via foliar Zn application.

At the soil chemistry level, the high alkalinity of iron-based materials (pH 10.9) elevates soil pH, thereby increasing the negative charge density in the soil matrix. This enhances the adsorption capacity for Cd^2+^ and Pb^2+^, while facilitating the formation of insoluble precipitates such as Fe/Mn oxides with these heavy metal ions (Luo et al., 2025; Chen et al., 2025a,d). Pearson correlation analysis confirmed a significant positive correlation (*P* ≤ 0.05) between elevated pH and Fe/Mn oxide-bound Cd fractions, verifying that pH-driven speciation transformation constitutes the core mechanism of Cd passivation (Yang et al., 2023; Zhou et al., 2024). This likely explains the 389.1% increase in Fe/Mn oxide-bound Cd and the 5.3% rise in residual Cd fractions. However, the Fe/Mn oxide-bound Pb fraction increased by only 29.6%, potentially due to competitive inhibition from soil organic matter (Wang S. et al., 2024). With no significant conversion to residual fractions, the passivation efficiency for Pb remained lower than for Cd, revealing differential immobilization mechanisms for Cd and Pb by the material.

### 4.2 ISF optimize the structure and function of bacterial communities

At the microbiological level, we further found that the ISF treatment effectively restructured the soil microbial community through targeted reconstruction, facilitating efficient bio-immobilization of Cd and Pb. Although the alpha diversity indices showed no significant differences (Supplementary Table 2), the ISF group contained 187 unique OTUs at the operational taxonomic unit level. Simultaneously, significantly divergent beta diversity was observed (*R*^2^ = 0.27, *P* = 0.02). These findings collectively demonstrated the successful restructuring of the microbial community composition (Figures 6A,B). Analysis of the community structure (Figures 6C–E) revealed specific enrichment of *Desulfobacterota* (implicated in heavy metal transformation) and *Nitrospirota* (involved in nitrogen cycling) (Bell et al., 2022; Zhang et al., 2025; Zhao et al., 2025). This enrichment was concurrent with significant suppression of *Actinobacteriota* (indicator taxa for low-disturbance environments) and *Acidobacteriota* (acid-sensitive phyla) (Das et al., 2021; Mitra et al., 2022). This shift signifies targeted selection of functional microbiota endowed with heavy metal resistance. Specifically, the abundance of *Desulfobacterota* exhibited a significantly negative correlation with DTPA-extractable Cd/Pb levels (Figure 7). This confirms its role in reducing SO4^2–^ to S^2–^/HS^–^ via the sulfate respiration pathway. Thereby, it generates CdS and PbS precipitates for heavy metal immobilization (Bell et al., 2022; Dyksma and Pester, 2024). Concurrently, this process activated the soil enzyme system (S-UE, S-AKP, S-DHA), significantly enhancing organic matter metabolic efficiency (Cui et al., 2017; Jat et al., 2020). Furthermore, ISF treatment substantially enhanced iron respiration (*P* < 0.01). The Fe(III) reduction to Fe(II) mediated by iron-reducing bacteria facilitated heavy metal coprecipitation (Liu et al., 2025; Shang et al., 2024), establishing a “sulfide-hydroxide dual immobilization barrier” (Sun et al., 2025).

Notably, microbial interaction network reconstruction further enhanced remediation sustainability (Figure 9). ISF treatment augmented network complexity and functional synchronicity (Huang et al., 2024). This established modular architectures with resilient buffering capacity (Fan et al., 2018). The coefficient of determination (R^2^) increased dramatically to 0.821, representing a 40-fold rise over the control (*R*^2^ = 0.021). Such optimization enabled microbial communities to maintain niche differentiation. Concurrently, it substantially strengthened system resilience against disturbances (Chen et al., 2024). These effects collectively provide critical micro-ecological assurance for sustained heavy metal immobilization.

### 4.3 ISF inhibits Cd and Pb transport to rice grains through synergistic regulation

At the plant translocation level, foliar application of Zn was performed. This reduced specific translocation factors for Cd and Pb in rice plants (Supplementary Figure 3). The reduction occurred to varying degrees. Ultimately, Cd and Pb accumulation in rice grains decreased. Cd^2+^ and Zn^2+^ have similar outer electron configurations. This similarity confers analogous chemical properties (Cao et al., 2024). When rice roots absorb metal ions from soil, an antagonistic relationship exists between Zn^2+^ and Cd^2+^ within rice plants. Increased Zn^2+^ levels may reduce binding sites of transmembrane transporters in roots (Wu et al., 2024). Consequently, this inhibits Cd^2+^ translocation to grains. Additionally, foliar Zn application may partially suppress transpiration. This consequently influences Cd absorption, translocation, and redistribution in rice (Rizwan et al., 2019). As an essential cofactor for key enzymes, Zn enhances photosynthetic efficiency and stress resistance (Jiang et al., 2023; Gao et al., 2025; Guo et al., 2024). Simultaneously, sulfur metabolism co-regulates Zn transporter expression (Dai et al., 2025), driving a 311.8% increase in Zn accumulation in rice grains (Figure 1C). These synergistic mechanisms collectively elucidate the superior remediation efficacy of ISF over IBS/FBA, providing an innovative and efficient solution for safe utilization of contaminated farmland.

## 5 Conclusion

This study found that the iron-based, sulfur-rich material combined with foliar Zn fertilizer (ISF) demonstrates significant synergistic benefits in remediating heavy metal contamination in paddy soils. Specifically, the ISF treatment increased rice biomass by 49.6%, while simultaneously reducing Cd and Pb concentrations in grains by 53.8 and 54.2%, respectively (both below national safety thresholds), and enhancing Zn accumulation by 311.8%. Concurrently, the bioavailable fractions of Cd and Pb in the soil decreased by 31.2 and 18.5%, respectively, whereas the Fe/Mn oxide-bound species increased by 389.1% (for Cd) and 29.6% (for Pb), with residual Cd showing a 5.3% increase. Notably, soil pH increased by 1.4 units, which was negatively correlated with heavy metal bioavailability, serving as a key immobilization mechanism. ISF also significantly improved the activities of critical enzymes (e.g., urease), increased total microbial OTU richness and 187 unique OTUs, while enriching key functional taxa including *Desulfobacteriota, Nitrospirota*, and *Geobacterales*. These changes promoted nitrogen fixation, sulfate/iron respiration and reinforced the stability of microbial interaction networks.

Future studies will clarify how the application of Zn to foliage enhances the efficacy of chelating Cd and Pb by recruiting Zn-adapted microbiota through the regulation of root exudates, while also examining their interactions with iron and sulfur-cycling microbial communities to promote the transformation into more stable, residual-like fractions with reduced bioavailability. It is important to note that these findings are based on short-term trials. The long-term remediation efficacy and potential ecotoxicological risks of the iron-based, sulfur-rich material (industrial solid waste) need to be validated through field trials.

## Data Availability

The original contributions presented in this study are included in this article/Supplementary material, further inquiries can be directed to the corresponding author.

## References

[B1] Bell E. Lamminmäki T. Alneberg J. Chen Q. Xiong W. L. Hettich R. L. (2022). Active anaerobic methane oxidation and sulfur disproportionation in the deep terrestrial subsurface. *ISME J.* 16 1583–1593. 10.1038/s41396-022-01207-w 35173296 PMC9123182

[B2] Bellotti G. Taskin E. Guerrieri M. C. Beone G. Menta C. Remelli S. (2022). Agronomical valorization of eluates from the industrial production of microorganisms: Chemical, microbiological, and ecotoxicological assessment of a novel putative biostimulant. *Front. Plant Sci.* 13:907349. 10.3389/fpls.2022.907349 35941943 PMC9356291

[B3] Cao K. Jaime-Pérez N. Mijovilovich A. Morina F. Bokhari Nadeem S. N. Liu Y. Q. (2024). Symplasmic and transmembrane zinc transport is modulated by cadmium in the Cd/Zn hyperaccumulator Sedum alfredii. *Ecotoxicol. Environ. Saf.* 275:116272. 10.1016/j.ecoenv.2024.116272 38564870

[B4] Chen L. J. Zhu G. F. Pascual-Garcia A. Dini-Andreote F. Zheng J. Wang X. Y. (2024). Unraveling the diversity dynamics and network stability of alkaline phosphomonoesterase-producing bacteria in modulating maize yield. *Imeta* 3:e260. 10.1002/imt2.260 39742308 PMC11683463

[B5] Chen S. F. Zhou Y. Q. Chen Y. R. Gu J. (2018). fastp: An ultra-fast all-in-one FASTQ preprocessor. *Bioinformatics* 34 884–i890. 10.1093/bioinformatics/bty560 30423086 PMC6129281

[B6] Chen T. Long S. L. Sun Y. Y. Zhong L. Shang C. M. Tang M. (2025c). Screening and human health risk assessment of rice varieties with cadmium/lead low accumulation. *J. Agro-Environ. Sci.* 44 1178–1189. 10.11654/jaes.2024-0667

[B7] Chen Y. L. Zhao X. Y. Zhang J. Wang H. H. Ye Z. L. Ma W. H. (2025d). Combined application of nitrate and schwertmannite promotes As(III) immobilization and greenhouse gas emission reduction in flooded paddy fields. *J. Environ. Chem. Eng.* 13:119845. 10.1016/j.jece.2025.119845

[B8] Chen Z. Gao H. Zhang J. Wang H. J. Ge L. Y. Mao R. T. (2025a). Making waves: Microbial-nitrate-zero valent iron/manganese synergy suppresses arsenic mobilization and greenhouse gas emissions in constructed wetlands. *Water Res.* 287:124492. 10.1016/j.watres.2025.124492 40902387

[B9] Chen Z. Zhao X. Y. Zeng S. T. Wang H. H. Ge L. Y. Zhang J. (2025b). Dual inhibition of As(III) mobilization and greenhouse gas emission achieved by photocatalytic birnessite-nitrate amendments in flooded paddy soils. *J. Environ. Chem. Eng.* 13:118011. 10.1016/j.jece.2025.118011

[B10] Cui H. B. Yang X. Xu L. Fan Y. Yi Q. T. Li R. Y. (2017). Effects of goethite on the fractions of Cu, Cd, Pb, P and soil enzyme activity with hydroxyapatite in heavy metal-contaminated soil. *RSC Adv.* 7:586945877. 10.1039/c7ra08786a

[B11] Dai L. H. Xie Z. Q. Ai T. X. Jiao Y. S. Lian X. Y. Long A. (2025). Zinc finger transcription factors BnaSTOP2s regulate sulfur metabolism and confer Sclerotinia sclerotiorum resistance in Brassica napus. *J. Integr. Plant Biol.* 67 101–116. 10.1111/jipb.13801 39503196

[B12] Das S. Kim G. W. Lee J. G. (2021). Silicate fertilization improves microbial functional potentials for stress tolerance in arsenic-enriched rice cropping systems. *J. Hazard. Mater.* 417:125953. 10.1016/j.jhazmat.2021.125953 33984783

[B13] Dyksma S. Pester M. (2024). Growth of sulfate-reducing Desulfobacterota and Bacillota at periodic oxygen stress of 50% air-O2 saturation. *Microbiome* 12:191. 10.1186/s40168-024-01909-7 39367500 PMC11451228

[B14] Fan K. K. Weisenhorn P. Gilbert J. A. Gilbert J. A. Chuet H. (2018). Wheat rhizosphere harbors a less complex and more stable microbial co-occurrence pattern than bulk soil. *Soil Biol. Biochem.* 125 251–260. 10.1016/j.soilbio.2018.07.022

[B15] Feng Q. F. Su S. M. Zhu Q. H. Zhang N. Yang Z. G. Zeng X. B. (2022). Simultaneous mitigation of Cd and as availability in soil-rice continuum via the addition of an Fe-based desulfurization material. *Sci. Total Environ.* 812:152603. 10.1016/j.scitotenv.2021.152603 34953852

[B16] Gao S. Tang X. Zhang J. Zhou Q. Y. Liu Q. Xu F. (2025). Zinc-selenium interaction regulates leaf photosynthesis and mediates grain sugar metabolism to improve the yield and quality of hybrid rice: A physiological perspective. *Plant Physiol. Biochem.* 221:109611. 10.1016/j.plaphy.2025.109611 39983599

[B17] Gao X. Peng Y. T. Zhou Y. Y. Adeel M. Chen Q. (2019). Effects of magnesium ferrite biochar on the cadmium passivation in acidic soil and bioavailability for packoi (Brassica chinensis L.). *J. Environ. Manag.* 251:109601. 10.1016/j.jenvman.2019.109610 31585274

[B18] Guo S. Q. Hu X. G. Wang Z. X. Yu F. B. Hou X. Xing B. S. (2024). Zinc oxide nanoparticles cooperate with the phyllosphere to promote grain yield and nutritional quality of rice under heatwave stress. *Proc. Natl. Acad. Sci. U S A.* 121:e2414822121. 10.1073/pnas.2414822121 39495932 PMC11573674

[B19] Huang Y. H. Yang Y. J. Li J. Y. Lü H. X. Zhao H. M. Xiang L. (2024). Root-associated bacteria strengthen their community stability against disturbance of antibiotics on structure and functions. *J. Hazard. Mater.* 465:133317. 10.1016/j.jhazmat.2023.133317 38218031

[B20] Jat H. S. Choudhary M. Datta A. Yadav A. K. Meena M. D. Devi R. (2020). Temporal changes in soil microbial properties and nutrient dynamics under climate smart agriculture practices. *Soil Tillage Res.* 199:104595. 10.1016/j.still.2020.104595 32362695 PMC7074002

[B21] Jiang Y. Wei C. Jiao Q. J. Li G. Z. Alyemeni M. N. Ahmad P. (2023). Interactive effect of silicon and zinc on cadmium toxicity alleviation in wheat plants. *J. Hazard. Mater.* 458:131933. 10.1016/j.jhazmat.2023.131933 37421854

[B22] Kong A. H. Wu J. K. (2012). Research advances for soil hydrolase activity in China. *Mod. Agric. Sci. Technol.* 16 251–260.

[B23] Lan W. Huang Y. F. Huang L. X. Huang Q. Li Y. L. Li X. (2020). Combined biochar and soda residues increases maize yields and decreases grain Cd/Pb in a highly Cd/Pb-polluted acid Udults soil. *Agric. Ecosyst. Environ.* 306:107198. 10.1016/j.agee.2020.107198

[B24] Lasisi A. Kumaragamage D. Casson N. Nora J. C. Inoka A. Srimathie P. I. (2023). Evaluating fall application of soil amendments to mitigate phosphorus losses during spring snowmelt. *Catena* 223:106908. 10.1016/j.catena.2022.106908

[B25] Li J. R. Xu Y. G. Liang X. F. Liu X. X. Jin C. Y. (2014). In situ immobilization remediation of heavy metals in contaminated soils: A review. *Ecol. Environ. Sci.* 23 721–728. 10.16258/j.cnki.1674-5906.2014.04.013

[B26] Li L. Li Q. Xiao A. Li C. Li Y. (2025). Regulation of soil properties by amendments and their impact on Cd fractions and bacterial community structure: Exploring the mechanism of inhibition on Cd phytoavailability. *Ecotoxicol. Environ. Saf.* 294:118033. 10.1016/j.ecoenv.2025.118033 40107216

[B27] Liu X. Li P. Bao K. Wang Y. Q. Wang H. L. Wang Y. H. (2025). Synergistic interaction between microbial nitrogen fixation and iron reduction in the environment. *ISME J.* 19:wraf212. 10.1093/ismejo/wraf212 40981677 PMC12516963

[B28] Liu Z. G. Xia Y. Meng Y. H. Cui S. H. Guan C. Y. (2021). Research advances in biomass-based carbon materials for remediation of heavy metal contaminated soil: Immobilization mechanism and analysis of related studies. *Chi. J. Environ. Eng.* 15 1140–1148. 10.12030/j.cjee.202012051

[B29] Lu G. Feng Z. Xu Y. Jin Y. Y. Zhang G. H. Hu J. H. (2023). Impact of phosphogypsum application on fungal community structure and soil health in saline–alkali-affected paddy fields. *Agronomy* 13:2726. 10.3390/agronomy13112726

[B30] Luo P. F. Sun Y. Xu Z. Q. Sun M. Rao Z. Q. Zhang N. (2025). Application of Fe-based S-rich material mitigates As and Cd toxicity in rice by alleviating root tip structural damage and improving bacterial community function. *J. Environ. Manage* 376:124429. 10.1016/j.jenvman.2025.124429 39923631

[B31] Lyu H. H. Zhao H. Tang J. C. Gong Y. Y. Huang Y. H. Wu Q. H. (2018). Immobilization of hexavalent chromium in contaminated soils using biochar supported nanoscale iron sulfide composite. *Chemosphere* 194 360–369. 10.1016/j.chemosphere.2017.11.182 29223115

[B32] Magoč T. Salzberg S. L. (2011). FLASH: Fast length adjustment of short reads to improve genome assemblies. *Bioinformatics* 27 2957–2963. 10.1093/bioinformatics/btr507 21903629 PMC3198573

[B33] Meng K. Wu X. Zhang X. Su S. Huang Z. Min X. (2019). Efficient adsorption of the Cd(II) and As(V) using novel adsorbent ferrihydrite/manganese dioxide composites. *ACS Omega* 4 18627–18636. 10.1021/acsomega.9b02431 31737822 PMC6854822

[B34] Mitra D. Mondal R. Khoshru B. Senapati A. Radha T. Mahakur B. (2022). Actinobacteria-enhanced plant growth, nutrient acquisition, and crop protection: Advances in soil, plant, and microbial multifactorial interactions. *Pedosphere* 32 149–170. 10.1016/s1002-0160(21)60042-5

[B35] Rieder S. R. Frey B. (2013). Methyl-mercury affects microbial activity and biomass, bacterial community structure but rarely the fungal community structure. *Soil Biol. Biochem.* 64 164–173. 10.1016/j.soilbio.2013.04.017

[B36] Rizwan M. Ali S. Zia Ur Rehman M. Adrees M. Arshad M. (2019). Alleviation of cadmium accumulation in maize (Zea mays L.) by foliar spray of zinc oxide nanoparticles and biochar to contaminated soil. *Environ. Pollut.* 248 358–367. 10.1016/j.envpol.2019.02.031 30818115

[B37] Rodrigues S. Avellan A. Bland G. D. Romeiro Miranda M. C. Larue C. Wagner M. (2024). Effect of a zinc phosphate shell on the uptake and translocation of foliarly Applied ZnO nanoparticles in pepper plants (Capsicum annuum). *Environ. Sci. Technol.* 58 3213–3223. 10.1021/acs.est.3c08723 38340051 PMC10882962

[B38] Schloss P. D. Westcott S. L. Ryabin T. (2009). Introducing mothur: Open-source, platform-independent, community-supported software for describing and comparing microbial. *Appl. Environ. Microbiol.* 75 7537–7541. 10.1128/AEM.01541-09 19801464 PMC2786419

[B39] Segata N. Izard J. Waldron L. (2011). Metagenomic biomarker discovery and explanation. *Genome Biol.* 12:R60. 10.1186/gb-2011-12-6-r60 21702898 PMC3218848

[B40] Shang C. M. Geng Z. X. Sun Y. Y. Che D. X. Zhao Q. J. Chen T. (2024). Lanthanum-modified phosphogypsum red mud composite for the co-adsorption of cadmium and arsenic: Mechanism study and soil remediation. *Agric. Basel* 14:464. 10.3390/agriculture14030464

[B41] Siczek A. Lipiec J. (2016). Impact of faba bean-seed rhizobial inoculation on microbial activity in the rhizosphere soil during growing season. *Int. J. Mol. Sci.* 17:784. 10.3390/ijms17050784 27213363 PMC4881600

[B42] Stefanowicz A. M. Niklińska M. Laskowski R. (2010). Metals affect soil bacterial and fungal functional diversity differently. *Environ. Toxicol. Chem.* 27 591–598. 10.1897/07-288.1 17944550

[B43] Sun Y. J. Fu M. Ang Y. Zhu L. N. He Y. Zeng H. L. (2022). Combined analysis of transcriptome and metabolome reveals that sugar, lipid, and phenylpropane metabolism are essential for male fertility in temperature-induced male sterile rice. *Front. Plant Sci.* 13:945105. 10.1016/j.watres.2024.122834 35968120 PMC9370067

[B44] Sun Y. Wu Z. Y. Lan J. R. Liu Y. Du Y. J. Ye H. P. (2025). Effect of sulfate-reducing bacteria (SRB) and dissimilatory iron-reducing bacteria (DIRB) coexistence on the transport and transformation of arsenic in sediments. *Water Res.* 270:122834. 10.3389/fpls.2022.945105 39608159

[B45] Tang B. Xu H. P. Song F. Q. Ge H. G. Yue S. Y. (2022). Effects of heavy metals on microorganismsand enzymes in soils of lead–zinc tailing ponds. *Environ. Res.* 207:112174. 10.1016/j.envres.2021.112174 34637758

[B46] Tóth G. Hermann T. Da Silva M. R. Montanarella L. (2016). Heavy metals in agricultural soils of the European Union with implications for food safety. *Environ. Int.* 88 299–309. 10.1016/j.envint.2015.12.017 26851498

[B47] Umar A. W. Naeem M. Hussain H. Ahmad N. Xu M. (2025). Starvation from within: How heavy metals compete with essential nutrients, disrupt metabolism, and impair plant growth. *Plant Sci.* 353:112412. 10.1016/j.plantsci.2025.112412 39920911

[B48] Wan X. M. Li C. Y. Parikh S. J. (2022). Simultaneous removal of arsenic, cadmium, and lead from soil by iron-modified magnetic biochar. *Environ. Pollut.* 261:114157. 10.1016/j.envpol.2020.114157 32086161

[B49] Wang F. Zhang J. Hu J. H. Wang H. J. Zeng Y. Q. Wang Y. H. (2024). Simultaneous suppression of As mobilization and N 2 O emission from NH 4 + /As-rich paddy soils by combined nitrate and birnessite amendment. *J. Hazard. Mater.* 465:133451. 10.1016/j.jhazmat.2024.133451 38228004

[B50] Wang G. S. Gao Q. Yang Y. F. Hobbie S. E. Reich P. B. Zhou J. Z. (2021). Soil enzymes as indicators of soil function: A step toward greater realism in microbial ecological modeling. *Glob. Change Biol.* 28 1935–1950. 10.1111/gcb.16036 34905647

[B51] Wang J. Riaz M. Babar S. Wang X. J. Xia X. Y. Yan B. H. (2025). Iron-modified biochar alleviates salt-alkali stress in cotton by enhancing iron availability and rhizosphere microbiome-metabolite interactions. *Plant Soil* 10.1007/s11104-025-07854-0 [Epub ahead of print].

[B52] Wang M. Mu C. Y. Lin X. Y. Ma W. Y. Wu H. T. Si D. F. (2024). Foliar application of nanoparticles reduced Cadmium content in Wheat (Triticum aestivum L.) grains via long-distance “leaf-root-microorganism” regulation. *Environ. Sci. Technol.* 58 6900–6912. 10.1021/acs.est.3c10506 38613493

[B53] Wang Q. (2007). Naive Bayesian classifier for rapid assignment of rRNA sequences into the new bacterial taxonomy. *Appl. Environ. Microbiol.* 73:16. 10.1128/AEM.00062-07 17586664 PMC1950982

[B54] Wang S. Gao W. Ma Z. Zhu Z. K. Luo Y. Wei L. (2024). Iron mineral type controls organic matter stability and priming in paddy soil under anaerobic conditions. *Soil Biol. Biochem.* 197:109518. 10.1016/j.soilbio.2024.109518

[B55] Wang X. J. Zhang D. M. Zhao S. T. Su Q. Lv J. L. Dai Y. C. (2024). Safe utilization effect of passivator, foliar Inhibitor, and their combined application on Cadmium-contaminated farmland. *Environ. Sci.* 45 7237–7244. 10.13227/j.hjkx.202312232 39628187

[B56] Wei S. Liu X. Tao Y. Wang X. Lin Z. Zhang Y. (2025). Strategy for enhanced soil lead passivation and mitigating lead toxicity to plants by biochar-based microbial agents. *J. Hazard. Mater.* 489:137512. 10.1016/j.jhazmat.2025.137512 39986095

[B57] Wen Y. B. Li W. Yang Z. F. Zhang Q. Z. Ji J. F. (2020). Enrichment and source identification of Cd and other heavy metals in soils with high geochemical background in the Knars region, Southwestern China. *Chemosphere* 245:125620. 10.1016/j.chemosphere.2019.125620 31869671

[B58] Wu Q. G. Liu Q. Q. Liu X. S. Zhao H. Wang X. F. Qin Y. H. (2024). Effect of in situ immobilization remediation combined with foliar spraying of ZnO on transfer and accumulation of Cd in different wheat varieties in weakly Alkaline soil. *Soils* 56 1311–1318. 10.13758/j.cnki.tr.2024.06.019

[B59] Xia F. Zhao Z. Niu X. Wang Z. (2024). Integrated pollution analysis, pollution area identification and source apportionment of heavy metal contamination in agricultural soil. *J. Hazard. Mater.* 465:133215. 10.1016/j.jhazmat.2023.133215 38101021

[B60] Yang L. Y. Wen T. T. Wang L. P. Takahiro M. Hao B. Lu X. (2019). The stability of the compounds formed in the process of removal Pb(II), Cu(II) and Cd(II) by steelmaking slag in an acidic aqueous solution. *J. Environ. Manage.* 231 41–48. 10.1016/j.jenvman.2018.10.028 30326337

[B61] Yang P. J. Jiang T. Cao D. Sun T. R. Liu G. L. Guo Y. Y. (2023). Unraveling multiple pathways of electron donation from phenolic moieties in natural organic matter. *Environ. Sci. Technol.* 57 16895–16905. 10.1021/acs.est.3c05377 37870506

[B62] Yang X. Zhang Z. X. Yuan Y. Z. Wang K. Y. Chen Y. Wang H. Y. (2022). Control efficiency of hexaconazole-lentinan against wheat sharp eyespot and wheat crown rot and the associated effects on rhizosphere soil fungal community. *Front. Microbiol.* 13:1014969. 10.3389/fmicb.2022.1014969 36212818 PMC9537369

[B63] Yin D. X. Wang X. Peng B. Tan C. Y. Lena Q. (2017). Effect of biochar and Fe-biochar on Cd and As mobility and transfer in soil-rice system. *Chemosphere* 186 928–937. 10.1016/j.hemosphere.2017.07.126 28830065

[B64] Zhang J. Y. Zhou H. Gu J. C. Huang F. Yang W. J. Wang S. L. (2020). Effects of nano-Fe_3_O_4_-modified biochar on iron plaque formation and Cd accumulation in rice (Oryza sativa L.). *Environ. Pollut.* 260:113970. 10.1016/j.envpol.2020.113970 32014742

[B65] Zhang M. M. He T. X. Wu P. Wang C. R. Zheng C. X. (2025). Recent advances in the nitrogen cycle involving actinomycetes: Current situation, prospect and challenge. *Bioresour. Technol.* 419:132100. 10.1016/j.biortech.2025.132100 39848446

[B66] Zhang T. Sun Y. F. Parikh S. J. Colinet G. Garland G. Huo L. J. (2024). Water-fertilizer regulation drives microorganisms to promote iron, nitrogen and manganese cycling: A solution for arsenic and cadmium pollution in paddy soils. *J. Hazard. Mater.* 477:135244. 10.1016/j.jhazmat.2024.135244 39032176

[B67] Zhao X. Chen Y. Hu J. Wang H. Ye Z. Zhang J. (2025). Efficacy of nitrate and biochar@birnessite composite microspheres for simultaneous suppression of As(III) mobilization and greenhouse gas emissions in flooded paddy soils. *Environ. Res.* 279:121757. 10.1016/j.envres.2025.121757 40324616

[B68] Zhong S. X. Li X. M. Fang L. P. Bai J. H. Gao R. C. Huang Y. H. (2024). Multifunctional roles of zinc in cadmium transport in soil-rice systems: Novel insights from stable isotope fractionation and gene expression. *Environ. Sci. Technol.* 58:12467. 10.1021/acs.est.4c01851 38966939

[B69] Zhou H. Wang Z. Y. Liu Y. Liu J. W. Gu J. F. Zeng P. (2021). Combined effects of soil amendment and Zinc fertilizer on accumulation and transportation of Cadmium in soil-rice system. *Environ. Sci.* 42 4452–4461. 10.13227/j.hjkx.202101150 34414745

[B70] Zhou Y. Cui Y. T. Yang J. Z. Chen L. Qi J. M. Zhang L. P. (2024). Roles of red mud in remediation of contaminated soil in mining areas: Mechanisms, advances and perspectives. *J. Environ. Manag.* 356:120608. 10.1016/j.jenvman.2024.120608 38508008

