# Supplementary material for: Synergistic remediation of Cd/Pb contamination in paddy soils using iron-based sulfur-rich material combined with foliar Zn fertilizer
Source: Front Microbiol. 2026 Jan 20;16:1756253. doi: 10.3389/fmicb.2025.1756253 (PMC12865293; doi:10.3389/fmicb.2025.1756253)
Supplement: Supplementary file 1 [file Supplementary_file_1.docx]

***Supplementary Material***

1. **Supplementary Figures and Tables**
   1. **Supplementary Figures**

l g soil + 8 mL MgCl_2_ (1 M), shock for 1 h (200 r/min), centrifuged for 10 min (4000r/min).

**EXC-****Cd/Pb**

①

**CBC-Cd/Pb**

① + 8 mLCH_3_COONa (1 M), shock for 1 h (200 r/min), centrifuged for 10 min.

②

**FMO-Cd/Pb Cd**

② + 20 mL CH_3_COOH (25%), water bathed (96±3℃) for 6 h, centrifuged for 10 min.

③

③ + 3 mL HNO_3_ (0.02 M) + 5 mL 30% H_2_0_2_, water bathed (85 ± 2 °C) for 2 h, + 3 mL 30% H_2_0_2_, water bathed (85 ± 2 °C) for 3 h, + 5 mL NH_4_OAc (3.2 M), shook for 0.5 h.

**OM-Cd/Pb**

④

**RES-Cd/Pb**

④ + HNO_3_-HF-HClO_4_ for digestion, filtration, and determination by ICP-MS.

**Supplementary Figure 1.** The detailed steps of the five-step continuous extraction process.


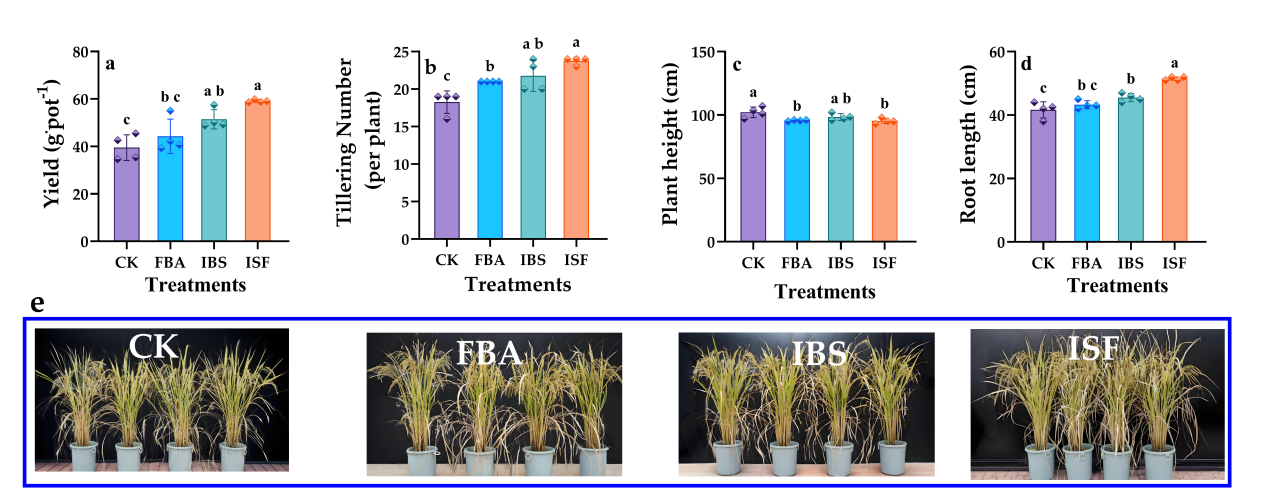


**Supplementary Figure 2.** Rice growth indices: (a) Yield, (b) Tillering number, (c) Plant height, (d) Root length, (e) Representative plant phenotypes (150-day growth). Distinct letter superscripts denote statistical significance (*P*<0.05). Data are expressed as mean ± SD (n = 4).


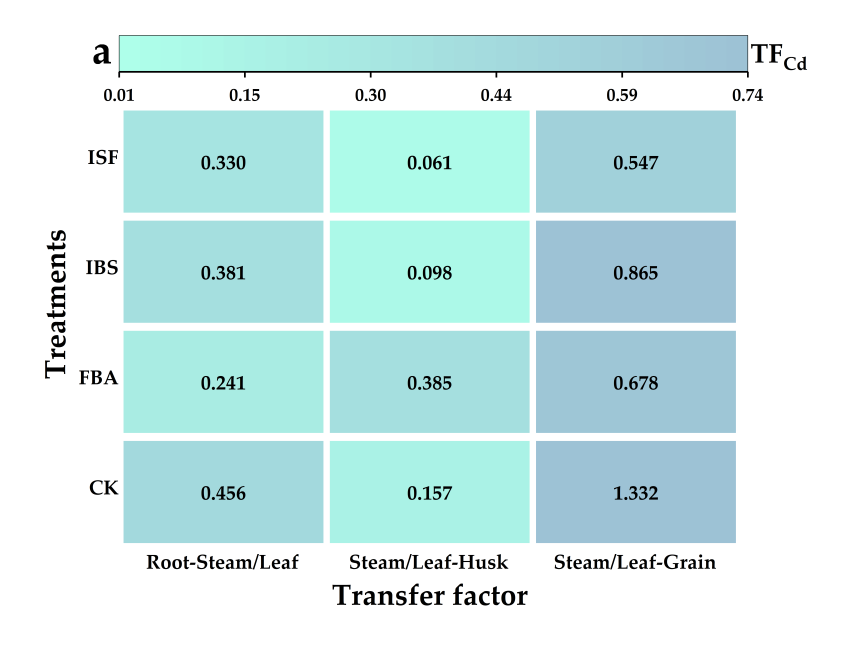


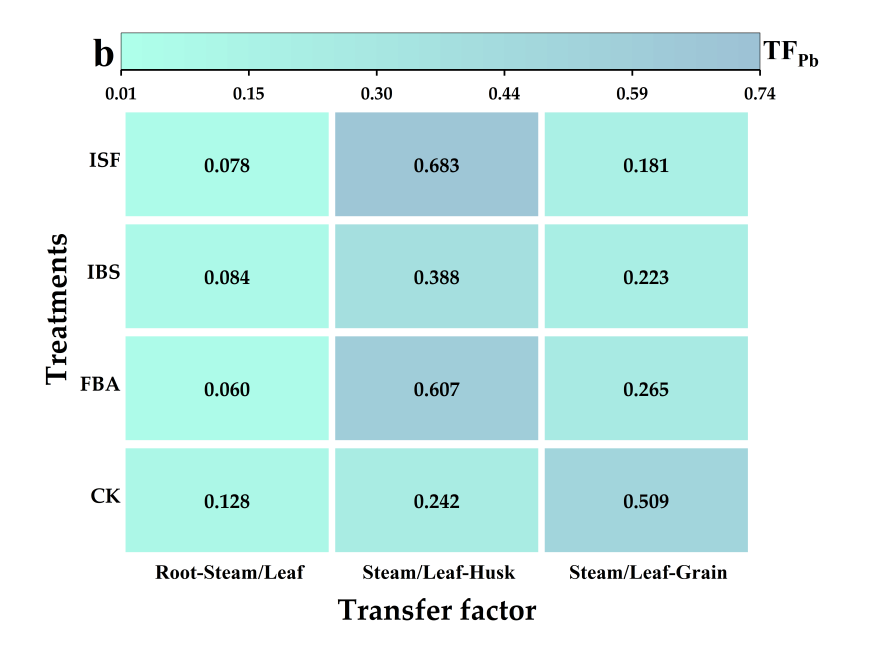


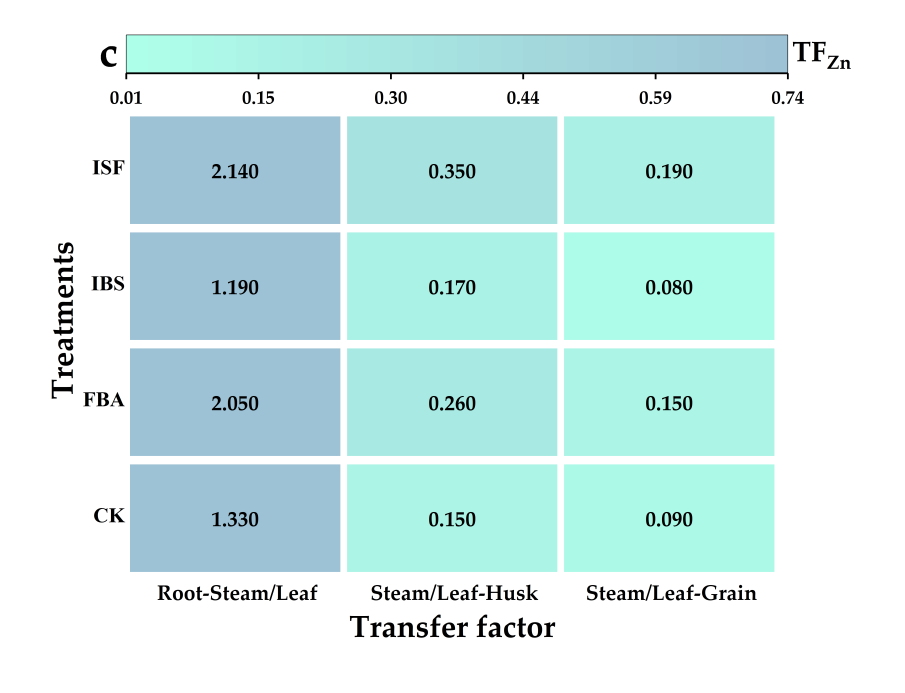


**Supplementary Figure 3.** Effects of FBA, IBS, and ISF amendments on the transfer factor (TF) of (a) Cd, (b) Pb, and (c) Zn in rice.


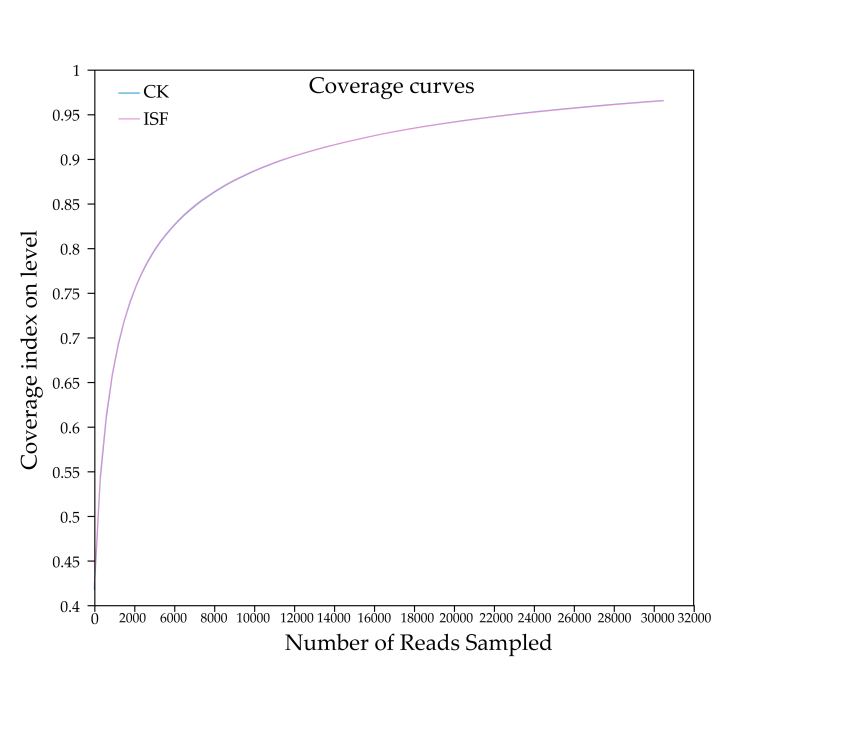


**Supplementary Figure 4.** Rarefaction based on the number of read and Coverage: the horizontal axis represents the amount of randomly sampled sequencing data, and the vertical axis represents the number of observed species.


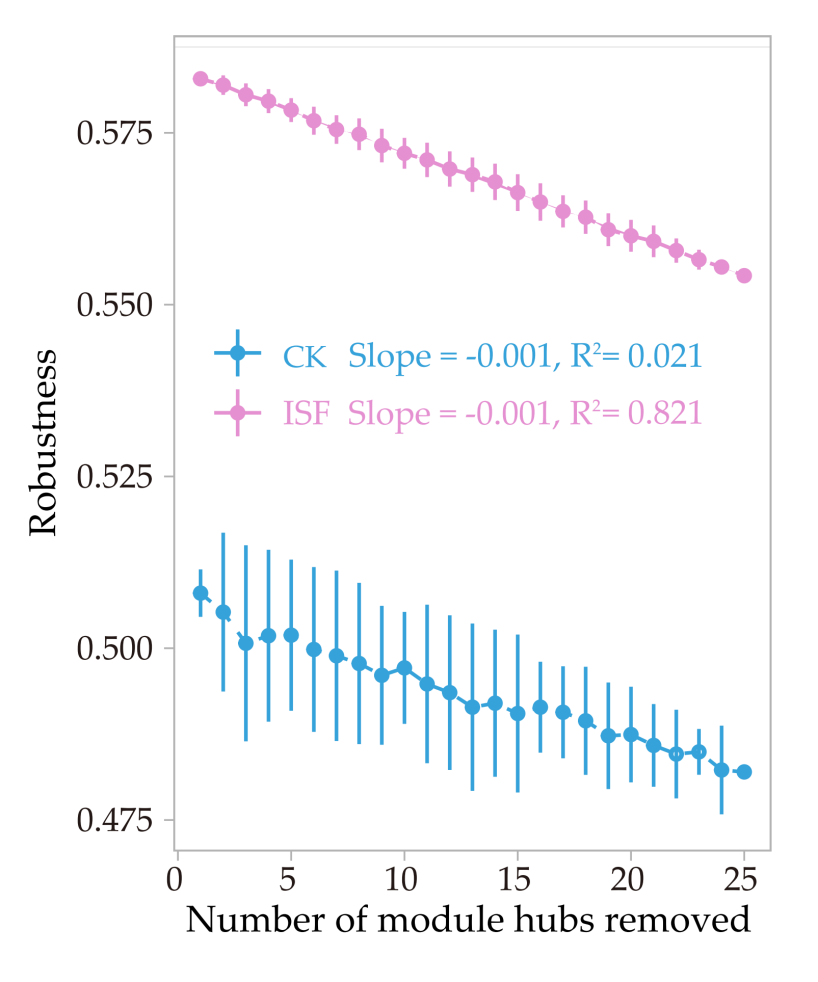


**Supplementary Figure 5.** Robust analysis of co-occurrence networks in bacterial communities.

- 1. **Supplementary Tables**

**Supplementary Table 1**. Physicochemical properties of the experimental soil.

| Properties | pH  （soil/water=1:2.5） | CEC  (g/kg) | SOM  （g/kg） | TN  (g/ kg) | TP  (g /kg) | TK  (g/kg) | Total Cd  (mg/kg) | Total Pb  (mg/kg) |
| --- | --- | --- | --- | --- | --- | --- | --- | --- |
| Values | 7.03 | 21.22 | 51.39 | 0.42 | 0.75 | 12.47 | 1.31 | 292..39 |

**Supplementary Table 2.** Changes in Alpha diversity index under the influence of ISF. Distinct letter superscripts denote statistical significance (*P*<0.05). Data are expressed as mean ± SD (n = 4).

| Treatment | Chao1 | Shannon | Simpson | Coverage |
| --- | --- | --- | --- | --- |
| CK | 3910.19±77.18a | 6.6916±0.07a | 0.0041±0.00062a | 0.9652±0.0012a |
| ISF | 3841.93±130.13a | 6.6575±0.06a | 0.0044±0.00580a | 0.9659±0.0014a |

**Supplementary Table 3.** Analysis of topological characteristics of co-occurrence networks of bacterial communities.

| Treatment | Node | Edge | | Modularity | Average clustering coefficient | Average path distance | Average degree | Network diameter | Graph density |
| --- | --- | --- | --- | --- | --- | --- | --- | --- | --- |
|  |  | Positive | Negative |  |  |  |  |  |  |
| CK | 344 | 1867  (49.77%) | 1888  (50.23%) | 0.899 | 1 | 1 | 21.808 | 1 | 0.064 |
| ISF | 371 | 2958  (55.97%) | 2327  (44.03%) | 0.854 | 1 | 1 | 28.491 | 1 | 0.077 |
